# Two Case Reports of Zoledronic Acid-Induced Acute Anterior Uveitis

**DOI:** 10.7759/cureus.72689

**Published:** 2024-10-30

**Authors:** David Alexander Vernaza Trujillo, Paola Milena Osorio Escorcia, Santiago Sierra Castillo, Monica Andrea Morales Garcia, Alin Abreu Lomba

**Affiliations:** 1 Epidemiology, Fundación Universitaria del Área Andina, Bogotá, COL; 2 Epidemiology and Public Health, Interinstitutional Group of Internal Medicine 1 (GIMI1) Universidad Libre, Cali, COL; 3 Endocrinology, New Clinic, Cali, COL; 4 Department of Medicine, CES University, Medellín, COL; 5 Epidemiology and Biostatistics, Clínica Imbanaco, Cali, COL; 6 Endocrinology, Clínica Imbanaco, Cali, COL

**Keywords:** acute anterior uveitis, bone densitometry, case report, osteoporosis, zoledronic acid

## Abstract

Zoledronic acid, or zoledronate, is a nitrogen-containing bisphosphonate widely used to treat osteoporosis and metastatic bone disease. It inhibits osteoclast function by binding to hydroxyapatite, reducing bone resorption and increasing bone mineral density. Despite its proven efficacy in increasing bone mineral density and reducing the incidence of fractures, adverse effects have been documented, including ocular side effects such as acute anterior uveitis. It's well known that osteoporosis primarily affects postmenopausal women and represents a significant economic burden in health care systems due to continued fragility fractures.

In this case series, we present two cases of postmenopausal women who have been diagnosed with severe osteoporosis. The first case is a 71-year-old woman with a history of rheumatoid arthritis, and the second is a 67-year-old patient with a history of congenital hip dysplasia. Both patients received IV zoledronic acid for osteoporosis treatment. Within a few days after infusion, both developed symptoms consistent with *Zoledronic acid*-induced *uveitis* (ZAIU), including ocular pain, redness, photophobia, and blurred vision.

Evaluations included bone densitometry, which confirmed severe osteoporosis, and phosphocalcic metabolism profiles-ocular symptoms, which led to ophthalmology consultations, where ZAIU was diagnosed through biomicroscopy and physical examination. Treatment included ophthalmic corticosteroids, with a posterior patient experiencing symptom resolution without sequelae. However, the occurrence of ZAIU led to a reconsideration of bisphosphonate therapy.

Although the incidence of ZAIU following zoledronic acid administration is low, it raises important considerations about managing osteoporosis in susceptible patients. The literature suggests vigilance for ocular side effects and a careful balance between the benefits and risks of bisphosphonate therapy.

## Introduction

Zoledronic acid, also known as zoledronate, is a nitrogen-containing bisphosphonate. These compounds exert an antiresorptive effect by inhibiting osteoclast function through their specific binding to hydroxyapatite at sites of high bone turnover, reducing bone absorption and increasing bone mineral density (BMD). Bisphosphonates are indicated for the treatment of osteoporosis, hypercalcemia of malignancy, Paget's disease, multiple myeloma, and metastatic bone cancer from solid tumors. Zoledronate is frequently prescribed to patients who are intolerant to oral bisphosphonates or who have metastatic bone disease because of its lower frequency of administration and higher potency. However, adverse reactions to this drug have been observed, including ocular side effects such as uveitis, scleritis, conjunctivitis, and iritis, especially after intravenous administration, which may be mediated by an acute phase reaction associated with interleukin-6 (IL-6) and tumor necrosis factor-alpha (TNF-α) [1,2].

Osteoporosis and fragility fractures mainly affect postmenopausal women, with significant economic costs in hospitalization, surgery, home care, disability, and death. The average life expectancy in Colombia is 78 years, and it is estimated that by 2050, the incidence of osteoporosis and, consequently, fragility fractures will increase significantly due to the aging of the population [3]. 

However, this scenario underscores the importance of evaluating the risks and benefits of pharmacological treatments, such as bisphosphonates, in postmenopausal women at high risk of fractures. Studies have shown the efficacy of these drugs in reducing fracture risk but also highlight the need to consider treatment rest periods to minimize associated risks, including ocular adverse effects [4].

This study is carried out under the CARE guidelines for case series.

## Case presentation

Case 1

The first case involves a 67-year-old woman with a significant past pathological history, including congenital hip dysplasia, which required bilateral total hip replacement, cataract in the right eye (OD), and mixed anxiety and depressive disorder in current management with escitalopram.

The diagnosis of primary osteoporosis was confirmed on 11/2022, with no history of fragility fractures. Bone densitometry (BDM) was reported on 11/2022, showing a BMD of lumbar spine L1-L4 of- 2.6 and 0.869 g/cm^2^ and forearm of -1.8 and 0.720 g/cm^2^. A phosphocalcium metabolism profile was also performed (09/2022), showing albumin levels of 4.42 g/dL, phosphorus of 4.5 mg/mL, parathyroid hormone (PTH) of 33.4 pg/mL, serum calcium of 9.48mg/dL, vitamin D of 33.19 ng/mL, and creatinine of 0.92 mg/dL. X-ray imaging (04/2023) of the dorsal lumbar and lumbosacral spine without evidence of fractures but with L5-S1 spondyloarthrosis (Table 1).

**Table 1:** Reference ranges for laboratory tests

Laboratory Test	Reference Range	Case 1	Case 2
Albumin (g/dL)	3.5 - 5.2 g/dL	4.42 g/dL	4.48 g/dL
Phosphorus (mg/dL)	2.5 - 4.5 mg/dL	4.5 mg/dL	4.4 mg/dL
Parathyroid Hormone (PTH)	15 - 65 pg/mL	33.48 pg/mL	26.6 pg/mL
Serum Calcium (mg/dL)	8.8 - 10.2 mg/dL	9.48 mg/dL	9.6 mg/dL
Urine Calcium (mg/day)	50 - 300 mg/day	-	80.64 mg/day
Vitamin D (ng/mL)	30 - 100 ng/mL	33.19 ng/mL	26.69 ng/mL
Serum Creatinine (mg/dL)	0.5 - 0,95 mg/dL	0.92 mg/dL	0.8 mg/dL

The treatment indicated by the geriatric service was zoledronic acid 5 mg IV and calcium citrate + vitamin D (1500 mg + 200 IU) one tablet daily. The application of Zoledronate infusion was performed on 01/2023 without immediate complications.

**Figure 1:**
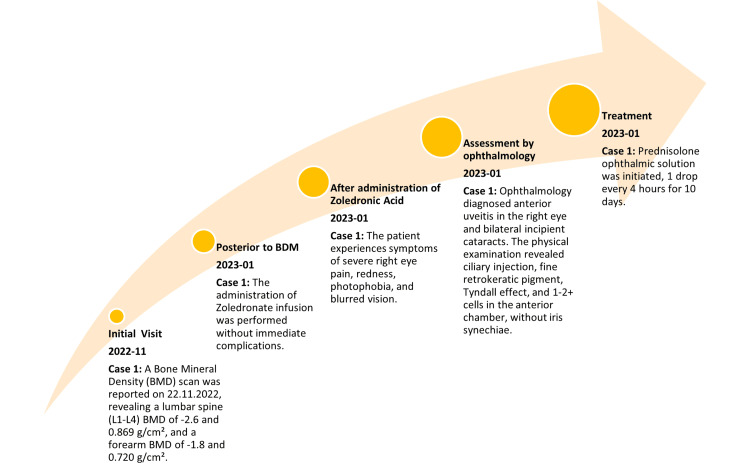
Timeline for case 1

Symptoms began two days after infusion (01/2023). Within 36 hours, the patient experienced high-intensity pain, redness, photophobia, and blurred vision in the right eye. She consulted an ophthalmologist, who diagnosed anterior uveitis in the right eye and bilateral incipient cataracts. Physical examination revealed ciliary injection, fine retrokeratotic pigment, Tyndall, and 1-2+ cells in the anterior chamber, with no iris synechiae. Anterior uveitis was managed with prednisolone ophthalmic solution, one drop every four hours for ten days, followed by gradual tapering. No ophthalmologic sequelae were observed after endocrinology evaluation in 01/2024 (Figure 1).

Case 2

The second case involves a 71-year-old woman with a pathological history of rheumatoid arthritis (diagnosed in 2021) under chronic steroid treatment (prednisolone, 2021-2022), arterial hypertension, chronic obstructive pulmonary disease (COPD), and supra ventricular tachycardia, which required double ablation in 2015 and 2017. One episode of cardiorespiratory arrest is mentioned without a specific date.

In February 2022, severe primary osteoporosis and vitamin D deficiency were diagnosed, with no history of fragility fractures. Bone densitometry (BMD) indicates a T-score of -4.1 at L1-L2 and -3.2 at the femoral neck, confirming advanced osteoporosis. In September 2023, the phosphocalcium metabolism profile showed albumin 4.48 g/dL, phosphorus 4.4 mg/dL, PTH 26.6 pg/mL, serum calcium 9.6 mg/dL, urine calcium 80.64 mg/day, vitamin D 26.69 ng/mL, and serum creatinine 0.8 mg/dL (Table 1).

Zoledronic acid 5 mg IV was administered in a single dose on 10/2023, and cholecalciferol 25,000 Units weekly was indicated after endocrinological evaluation.

**Figure 2:**
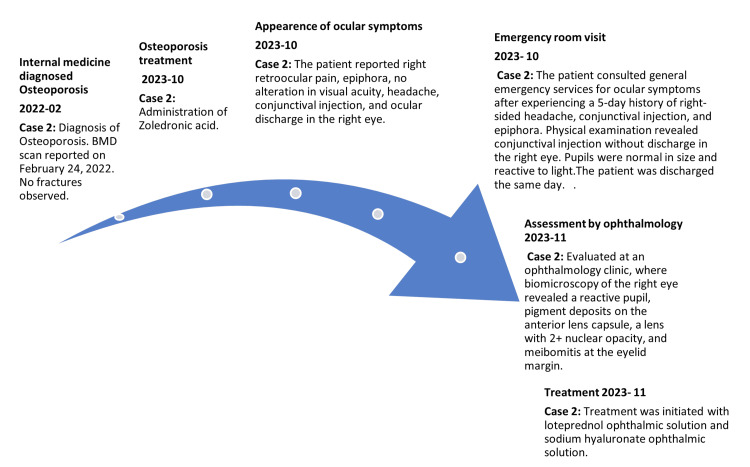
Timeline of case 2

The patient starts to experience symptoms of arthralgias and myalgias on 10/2023. She consulted the emergency department on 10/2023 for right eye pain, ocular discharge, headache, and epiphora without alteration of visual acuity. The eye physical examination showed conjunctival injection without discharge and photonormoreactive pupils. She was treated with Diclofenac 75 mg IM single dose, gentamicin eye drops, and a combination of neomycin, polymyxin B, and dexamethasone eye drops. A subsequent ophthalmologic evaluation on 11/2023 identifies anterior uveitis in the right eye and bilateral incipient cataracts. She started treatment with loteprednol in an ophthalmic solution and sodium hyaluronate in an ophthalmic solution. No sequelae were reported in subsequent controls (Figure 2).

## Discussion

Zoledronic acid is shown to significantly reduce the incidence of vertebral fractures by 62-70% (with a marked reduction at one year), hip fractures by 41%, and non-vertebral fractures by 21-25% (over three years) in patients with osteoporosis with prevalent vertebral fractures and/or osteoporosis as determined by hip bone low mineral density [5]. Both the Endocrine Society and the Bone and Health Osteoporosis Foundation (BHOF) recommend, in their guidelines, initial treatment with bisphosphonates (alendronate, risedronate or zoledronic acid) for patients at a high risk of fracture, criteria that were applied for the selection of treatment in the patients mentioned in our report, who happened to be two postmenopausal women over 50 years of age with severe osteoporosis according to BMD [6].

Nitrogen-containing bisphosphonates, such as Zoledronic acid, disrupt the mevalonate pathway, a crucial metabolic route for cholesterol and isoprenoid precursor synthesis. By blocking this pathway, there is an inhibition of the formation of farnesyl pyrophosphate (FPP) and geranylgeranyl pyrophosphate (GGPP), which are essential for the prenylation of small GTP-binding proteins that are crucial for osteoclast function and the survival. Consequently, osteoclast apoptosis occurs, leading to inhibited bone resorption, the primary therapeutic effect sought in conditions like osteoporosis and metastatic bone cancer. Administration of nitrogen-containing bisphosphonates can trigger an immune response led by gamma-delta T lymphocyte activation. These lymphocytes recognize non-peptidic antigens, such as isopentenyl pyrophosphate (IPP) and dimethylallyl pyrophosphate (DMAPP), accumulated due to mevalonate pathway inhibition [7-14].

Activated lymphocytes release proinflammatory cytokines like IL-1, IL-6, TNF-α, and interferon-gamma (IFN-γ). These cytokines increase local inflammation at the administration site and incite a self-systemic inflammatory response [14]. Gamma-delta T lymphocyte activation and subsequent cytokine release can induce inflammation in ocular structures, particularly the uvea, leading to uveitis characterized by symptoms such as ocular redness, pain, photophobia, and blurred vision. Besides local effects, immune activation can generate systemic symptoms like fever, pain, nausea, and fatigue, observed in about 25% of patients treated with nitrogen-containing bisphosphonates. Approximately 11% of patients may develop bilateral orbital conditions, strongly linked with more severe systemic symptoms [8-13].

Currently, an incidence of acute anterior uveitis following zoledronic acid application has been reported to be up to 1.1%, with an average time to symptom onset three days after de amino bisphosphonate infusion [7]. In 2013, an analysis of data from a randomized clinical trial in postmenopausal patients older than 65 years undergoing treatment for osteoporosis revealed that 8 of 1001 patients (0.8%) who received zoledronic acid treatment developed moderate to severe acute anterior uveitis within seven days after drug infusion [4,15]. In our patients' case, the uveitis presentation was consistent with that described in the literature, manifesting within the first seven days after drug administration [16,17].

A 2020 retrospective pharmacovigilance study based on adverse drug reactions reported in VigiBase, the WHO international pharmacovigilance database, entitled "Evolution of the spectrum of drug-induced uveitis in the era of immune checkpoint inhibitors", reports that of 1,404 cases corresponding to 37 drugs with a significant over-reporting signal, bisphosphonates accounted for 26.9% of patients. Of 48,990 safety reports, 195 were for zoledronic acid uveitis. Initial studies indicate a relative risk for acute anterior uveitis in the first application of bisphosphonates of 1.45 (95% CI: 1.25-1.68) [18].

Both cohort studies and analyses of clinical trial data showed that patients who developed zoledronic acid-induced uveitis were both women and men being treated for osteoporosis, and mostly in the case of women, postmenopausal. Our patients presented the development of the event similarly after the first application of zoledronic acid [11].

Reviewing the current literature, 34 case reports of zoledronic acid-induced uveitis were found. Most of these reports evidenced the appearance of unilateral symptoms within seven days after the application of a first dose of zoledronic acid, with a favorable response to treatment with ophthalmic corticosteroids and without subsequent sequelae, characteristics that were also observed in our case series [19-20].

The development of zoledronic acid-induced uveitis (ZAIU) is still a source debated as a contraindication for continuing treatment, without being mentioned as an absolute contraindication in most current guidelines for the management of osteoporosis [11]. This leads to the fact that, in routine practice and when a case is reported, changes in medication could be chosen. This decision is based on the favorable response to treatment with ophthalmic corticosteroids and the absence of sequelae in affected patients. In addition, to date, there are no data available on patient follow-ups that indicate or are clear regarding the occurrence of a new uveitis event in subsequent doses. This scenario highlights the importance of carefully evaluating the benefit-risk balance when considering the continuation of bisphosphonate therapy in patients who have developed ZAIU, underscoring the need for future research to guide clinical decisions more accurately in these cases [7].

Patient perspective

In the reported cases of patients who developed uveitis after receiving zoledronate, there was a reluctance to continue with future doses of the same drug, accompanied by concern about similar adverse reactions with other osteoporosis treatments. Both patients expressed a desire to explore therapeutic alternatives, expressing fear of possible sequelae associated with the adverse event, although they had no residual ophthalmologic sequelae.

## Conclusions

Zoledronic acid continues to be an effective and recommended treatment for the prevention of fractures in patients with osteoporosis; the association with acute anterior uveitis (AAUIU) poses a major clinical challenge. Although the incidence of this complication is low, its occurrence may lead to reconsidering the therapeutic regimen employed in each patient. It is crucial for physicians to be alert to symptoms of ZAIU after administration of zoledronic acid and to carefully evaluate the benefits and risks of continuing with the same treatment in those patients who present with this adverse reaction. The lack of an absolute contraindication in current guidelines gives rise to clinical judgment based on individual experience, patient-specific details, and personal pathological history.

Future research should focus on understanding the underlying mechanisms leading to ZAIU, identify risk factors for its development, and clarify the risk of recurrence with subsequent doses of bisphosphonates. In addition, develop clearer guidelines for the management of patients who have developed ZAIU, including recommendations on therapeutic alternatives and follow-up strategies that will lead to an Improvement of our understanding of ZAIU and optimizing the management of affected patients can ensure that the benefits of zoledronic acid treatment for osteoporosis are maximized while risks are minimized.
